# Increased C-Peptide Immunoreactivity in Insulin Autoimmune Syndrome (Hirata Disease) Due to High Molecular Weight Proinsulin

**DOI:** 10.1093/clinchem/hvab043

**Published:** 2021-05-24

**Authors:** Richard G Kay, Peter Barker, Keith Burling, Mark Cohen, David Halsall, Frank Reimann, Fiona M Gribble, Robert K Semple, David Church

**Affiliations:** 1 University of Cambridge Metabolic Research Laboratories, Wellcome Trust–MRC Institute of Metabolic Science, Cambridge, UK; 2 Core Biochemical Assay Laboratory, Cambridge University Hospitals NHS Foundation Trust, Cambridge, UK; 3 Department of Diabetes & Endocrinology, Royal Free London NHS Foundation Trust, London, UK; 4 Department of Clinical Biochemistry and Immunology, Cambridge University Hospitals NHS Foundation Trust, Cambridge, UK; 5 National Institute for Health Research Cambridge Biomedical Research Centre, Cambridge, UK; 6 University of Edinburgh Centre for Cardiovascular Science, Queen’s Medical Research Institute, Edinburgh, UK

## Abstract

**Background:**

Determination of C-peptide is important in the investigation of unexplained hyperinsulinemic hypoglycemia because a high C-peptide concentration usually indicates endogenous insulin hypersecretion. Insulin autoimmune syndrome (IAS) denotes hyperinsulinemic hypoglycemia due to insulin-binding antibodies that prolong insulin half-life. C-peptide clearance is considered to be unaffected, and although a marked C-peptide immunoreactivity in hypoglycemic samples has been reported, it has been suspected to be artifactual. High-resolution mass spectrometry enables examination of the basis of C-peptide-immunoreactivity in IAS.

**Methods:**

Precipitation of plasma with polyethylene glycol was followed by C-peptide immunoassay. Plasma peptides extracted by solvent precipitation were characterized by nano-LC–MS/MS and analyzed using an untargeted data-dependent method. Peptides related to proinsulin, in amino acid sequence, were identified using proprietary bioinformatics software and confirmed by repeat LC–MS/MS analysis. Gel filtration chromatography coupled to LC–MS/MS was used to identify proinsulin-related peptides present in IAS immunocomplexes. Results were compared with those from C-peptide immunoassay.

**Results:**

Polyethylene glycol precipitation of IAS plasma, but not control plasma, depleted C-peptide immunoreactivity consistent with immunoglobulin-bound C-peptide immunoreactivity. LC–MS/MS detected proinsulin and des 31,32 proinsulin at higher abundance in IAS plasma compared with control plasma. Analysis by gel filtration chromatography coupled to LC–MS/MS demonstrated proinsulin and des 31,32 proinsulin, but no C-peptide, in plasma immunocomplexes.

**Conclusions:**

Antibody binding can enrich proinsulin and des 31,32 proinsulin in IAS immunocomplexes. Proinsulin cross-reactivity in some C-peptide immunoassays can lead to artifactually increased C-peptide results.

## Introduction

Insulin autoimmune syndrome (IAS, or Hirata disease) denotes spontaneous hyperinsulinemic hypoglycemia due to insulin-binding autoantibodies in individuals not receiving insulin therapy (1, 2). Autoantibodies form immunocomplexes with insulin, sequestering acutely secreted insulin (3) and retarding its clearance (4), consequently maintaining inappropriate total plasma insulin concentrations compared with the contemporaneous blood glucose concentration. Suspicion of the disorder may arise following the finding of very high plasma insulin concentration with an increased insulin/C-peptide molar ratio in a hypoglycemic sample (5–7). Willful misadministration of exogenous insulin is sometimes confused with this profile and laboratory analysis assumes decisive importance in discriminating these entities.

**Fig. 1.**
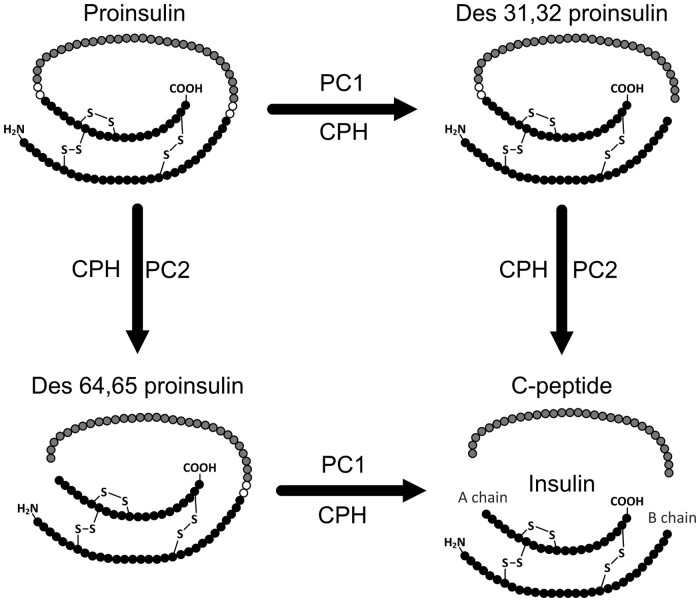
Proinsulin processing. Mature insulin is cleaved from proinsulin by the action of 3 peptidases: prohormone convertase 1 (PC1), prohormone convertase 2 (PC2), and carboxypeptidase H (CPH). The amino acid residues (○) are cleaved by proteases with preferential processing of the 31/32 site before the 64/65 site. C-peptide and insulin (A chain and B chain linked by 2 disulfide bonds) are secreted in equimolar amounts with comparatively very low amounts of the 2 des proinsulin forms being present in the plasma of healthy individuals.

Hyperinsulinemic hypoglycemia with high C-peptide usually indicates endogenous insulin hypersecretion (8). High C-peptide immunoreactivity has been reported in IAS, even in the face of frequent profound hypoglycemia (6, 7, 9–17). This has the potential to confuse managing clinicians since normal endogenous C-peptide production by the pancreas is inhibited in response to exogenous insulin-induced hypoglycemia (18, 19). However, immunoassay of C-peptide may yield artifactually increased results in IAS (7). The cause of this is not known. The cause of putative interference in C-peptide immunoassay is unconfirmed, although cross-reactivity of antibody-bound proinsulin has been postulated (10, 15); endoproteolytic cleavage of proinsulin and its intermediates des 64,65 proinsulin and des 31,32 proinsulin, generates (insulin and) C-peptide (Fig. 1). The amino acid sequence of C-peptide, a single polypeptide chain, is present in proinsulin, des 31,32 proinsulin, and des 64,65 proinsulin such that these precursors have the potential to cross-react in C-peptide immunoassay.

Identification of hormone immunocomplexes by immunoassay is challenging because autoantibody and assay reagents may compete to bind hormones, and the signal generated in plasma is unlikely to represent solely unbound or total (unbound plus bound) analyte. Immunocomplexes can be separated by precipitation, or by physical methods, such as gel filtration chromatography (GFC) (5–7), to discriminate high molecular weight (HMW, antibody-bound) and monomeric immunoreactivity. Mass spectrometry is an orthogonal method to quantify plasma peptides that is not reliant on immunoreactivity and exhibits far greater selectivity than immunoassay. Until recently, mass spectrometry has been limited by analytic sensitivity, but developments in both sensitivity and resolution of mass spectrometers have greatly enhanced parallel analysis of plasma peptides extracted from plasma by precipitation (20), solid-phase extraction (SPE) (21), or targeted extraction (22). Consequently, it is now possible to interrogate the composition of antibody-bound peptide complexes in IAS with far greater selectivity. Targeted LC–MS/MS analysis offers highly specific quantification of insulin and C-peptide (23, 24), while its untargeted application in combination with bioinformatic analysis can identify structurally similar peptides (20). In this study, we analyzed IAS plasma using LC–MS/MS with an untargeted peptidomic approach with the aim of identifying whether C-peptide immunoreactivity was attributable to cross-reacting proinsulin/proinsulin-derived molecules.

## Materials and Methods

### Patients and Blood Sampling

Studies were performed in accordance with the Declaration of Helsinki (2000). Heparinized plasma samples from 7 patients with IAS were evaluated for potential interference with a C-peptide immunoassay by the UK Severe Insulin Resistance Supra-Regional Assay Service, Cambridge University Hospitals NHS Foundation Trust, Cambridge. A summary of the clinical characteristics of patients studied, the investigations undertaken on initial presentation, and the case histories have been published (6, 7). Nonfasting blood samples were collected on ice, and plasma separated and frozen at −80 °C until analysis. Insulin and C-peptide immunoreactivity in nonfasting neat plasma, and insulin immunoreactivity in the same sample following a 1 in 5 and a 1 in 50 dilution with assay diluent, were determined using the DiaSorin LIAISON^®^ XL.

### Plasma Precipitation with Polyethylene Glycol

Polyethylene glycol (PEG) precipitation studies were performed as previously published (5), with PEG precipitation ratio (PPR) defined as the C-peptide immunoreactivity remaining in the supernatant following PEG precipitation compared with that in matched saline-diluted plasma, expressed as a percentage. To overcome a LIAISON XL sampling error with PEG supernatant (likely exacerbated by PEG-induced increased viscosity), a 1 in 2 0.9% (w/v) saline dilution of PEG supernatant was used.

### Untargeted Plasma Peptidomic Analysis

Untargeted peptidomic analysis of separated heparinized plasma, previously stored at −80 °C before being thawed on ice, was undertaken. 50 µL were transferred to an Eppendorf^®^ microcentrifuge tube and protein precipitated with 300 µL 80% (v/v) aqueous acetonitrile (ACN) solution before centrifugation at 12 000*g* for 10 min at 4 °C. The resulting supernatant was removed by aspiration and transferred to Eppendorf LoBind microcentrifuge tubes. Using a Biotage^®^ SPE Dry system, the solvent was evaporated under oxygen-free nitrogen heated to 40 °C. Samples were reconstituted in 200 µL of 0.1% (v/v) formic acid (FA), and SPE was performed using a Waters Oasis^®^ PRiME HLB 96-well µElution Plate as previously described (20). Eluate was evaporated and the residue reconstituted in 75 µL of 10 mmol/L dithiothreitol in 50 mmol/L ammonium bicarbonate, then heated for 60 min at 60 °C. 15 µL of 100 mmol/L iodoacetamide in 50 mmol/L ammonium bicarbonate was then added, and the solution left in the dark for 30 min before addition of 20 µL of 1% FA (v/v). 30 µL samples were injected into the LC–MS system (20). In brief, analysis employed a Thermo Scientific™ UltiMate™ 3000 RSLC–nano system coupled to a Thermo Scientific Q Exactive™ Plus. Peptides were captured on a 0.3 × 5 mm Thermo Scientific C18 PepMap100 peptide trap column for 15 min before switching in-line to a 0.075 × 250 mm Thermo Scientific EASY-Spray™ LC Column. Electrospray analysis was carried out using a spray voltage of 1.8 kV, and S-lens setting of 70 V. A full scan range of 400–1600 *m/z* was used with a resolution of 75 000, before the top 10 ions of each spectrum were selected for MS/MS analysis. Ions selected for fragmentation were added to an exclusion list for 30 s to prevent their repeated fragmentation.

Data were compared with the human Swissprot database (www.uniprot.org; downloaded 26-Oct-2017) using Bioinformatics Solutions Inc. PEAKS Studio v.8.5 (20). Mass spectra were matched against the database with a “no enzyme” setting, with fixed cysteine carbamidomethylation, N-terminal acetylation, N-terminal pyroglutamate, methionine oxidation, and C-terminal amidation as variable modifications. Results were filtered with a false positive discovery rate of 1% against a decoy database, and at least one unique peptide match was required. PEAKS software identified peptides of up to 65 amino acids in length.

### LC–MS/MS Characterization and Semiquantitative Analysis of Intact Proinsulin-Derived Peptides

To identify intact plasma proteins composed of the proinsulin-derived peptides identified in the peptidomic screen, plasma samples were reextracted and analyzed without reduction and alkylation, keeping disulfide bonds intact. 250  µL of plasma were transferred to a 2 mL 96-well plate, and 1 mL of aqueous 80% (v/v) ACN with 1 ng/mL bovine insulin internal standard was added then mixed for 20 s before centrifugation at 2900*g* for 10 min at 4 °C. The supernatant was removed and transferred to an Eppendorf LoBind plate and solvent evaporated to dryness under oxygen-free nitrogen. The residue was reconstituted in 200 µL 0.1% (v/v) aqueous FA, and SPE was performed as described above. Following elution, 75 µL 0.1% (v/v) FA was added to reduce the organic solvent prior to direct LC–MS/MS analysis.

Fifty microliters of sample extract were loaded at 300 µL/min onto a Waters ACQUITY UPLC HSS T3 Column (100 Å, 1.8 µm, 2.1 × 50 mm) heated to 60 °C. Starting conditions were 78% A [0.1% FA in water (v/v)) and 22% B (0.1% FA in ACN (v/v)], changing to 68% A and 32% B after 6.4 min. The column was washed with 90% B for 1.6 min before returning to the starting conditions for 2 min. MS was performed in positive electrospray mode with a needle voltage of 3 kV, gas flow settings of 55 and 10 for sheath and aux, respectively. Temperatures were set at 350 °C for the gas aux, and 350 °C for the transfer capillary, and the S-lens was set at 70 V. MS data were acquired from *m/z* 700–1600, with a resolution of 70 000 and an automatic gain control target of 3 × 10^6^ ions. LC–MS data were interrogated for ions with *m/z* ratios assigned to potential proinsulin-derived peptides.

To confirm ions attributed to putative proinsulin-derived peptides, targeted product ion scan analysis of extracts from an IAS plasma sample was performed. A more proinsulin-centric LC–MS/MS analysis was used: full scan analysis was performed from *m/z* 800–1600, and a parallel-reaction monitoring-based analysis was performed for proinsulin (*m/z* 1342.0), des 31,32 proinsulin (*m/z* 1300.0), des 31–33 proinsulin (*m/z* 1281.6), insulin (*m/z* 1162.3), and C-peptide (*m/z* 1007.7). To confirm identification of proinsulin, the WHO international proinsulin standard (09/296) was obtained from NIBSC and analyzed using LC–MS/MS. After the identities of proinsulin-derived peptides were established, their relative abundances were assessed in IAS and control plasma.

### Gel Filtration Chromatography with C-Peptide Immunoassay or LC–MS/MS

GFC of plasma from the same sample used above was undertaken using a previously described protocol (5), but adapted so that 3 mL fractions were collected [final volume 3.5 mL, 4% (w/v) BSA]. Fractions were analyzed for C-peptide using the DiaSorin LIAISON XL immunoassay.

GFC of plasma was then repeated on the same sample, with 3 mL fractions collected into 5 mL Eppendorf LoBind microcentrifuge tubes following addition of 10% (v/v) mouse plasma [5 µL, diluted in 0.1% (v/v) FA]. HMW and monomeric fractions chosen for MS analysis covered the GFC peaks demonstrable by C-peptide immunoassay. Prior to SPE, 500 µL 6 mol/L guanidine hydrochloride was added to each fraction to release antibody-bound peptides. Eluent was diluted with 75 µL 0.1% (v/v) FA, and 30 µL of this solution was analyzed by nano LC–MS/MS. A targeted parallel-reaction monitoring-based analysis was again performed for proinsulin (*m/z* 1342.0), des 31,32 proinsulin (*m/z* 1300.0), des 31–33 proinsulin (*m/z* 1281.6), and C-peptide (*m/z* 1007.7).

## Results

Immunoassay results (Table 1) demonstrated marked hyperinsulinemia in all patients, and dilution of plasma revealed a high insulin/C-peptide molar ratio with an increase in insulin immunoreactivity following correction for dilution, characteristic of insulin-binding antibodies (5–7).

**Table 1:** Insulin and C-peptide immunoreactivity in non-fasting plasma [adapted from Church et al. (6, 7)].

Patient number	1	2	3	4	5	6	7
Insulin (<60), pmol/L	>3000	>3000	>3000	2906	2781	1340	782
Insulin (diluted 1 in 5), pmol/L	>15 000	11 585	7020	5630	7805	3912	4601
Insulin (diluted 1 in 50), pmol/L	68 950	30 620	–	6605	12 100	5930	11 440
C-peptide (174–960), pmol/L	4960	5580	3750	3280	3110	1190	2380
Insulin/C-peptide (<1)	–	–	–	0.9	0.9	1.1	0.3
Insulin (diluted 1 in 5)/C-peptide (<1)	–	2.1	1.9	1.7	2.5	3.3	1.9
Insulin (diluted 1 in 50)/C-peptide (<1)	13.9	5.5	–	2.0	3.9	5.0	4.8

Insulin immunoreactivity was determined using the dilution factor following a 1 in 5 and a 1 in 50 dilution. Fasting reference limits are shown in brackets.

### Plasma Precipitation with Polyethylene Glycol

**Fig. 2.**
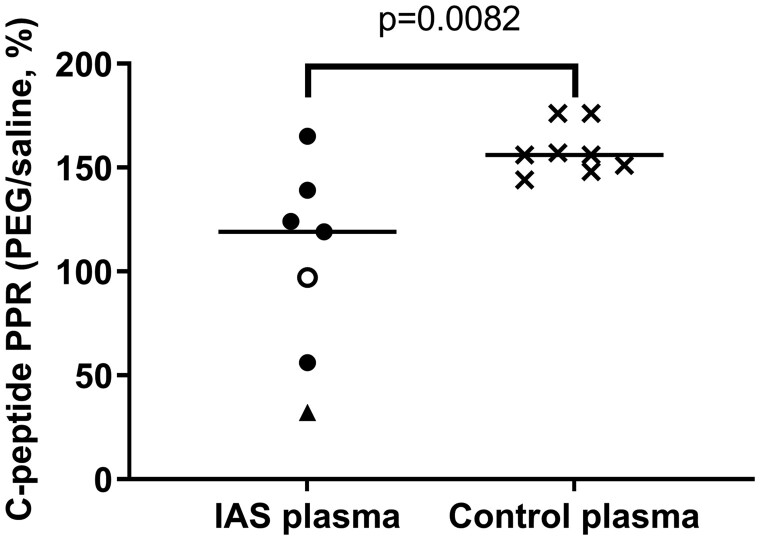
Polyethylene glycol precipitation ratio (PPR, PEG supernatant/saline, %) of C-peptide immunoreactivity of insulin autoimmune syndrome (IAS) and control plasma. For IAS, plasma median C-peptide PPR was 119% (interquartile range 56–139%). For control plasma, median C-peptide PPR was higher than IAS samples at 156% (149–171%) (Mann–Whitney test, *P* = 0.0082). IAS plasma represented as ○ was not used in mass spectrometry studies. IAS plasma represented as ▲ was used in gel filtration chromatography studies.

To identify HMW C-peptide immunoreactivity, PEG precipitation of 7 IAS plasma samples, and 8 control plasma samples with C-peptide concentrations spanning the DiaSorin LIAISON XL assay range, was undertaken (Fig. 2). The median IAS PPR was 119%, lower than the 156% median of control samples (Mann–Whitney test, *P* = 0.0082).

### Nano LC–MS/MS Analysis of Reduced and Alkylated Plasma Extracts

**Fig. 3.**
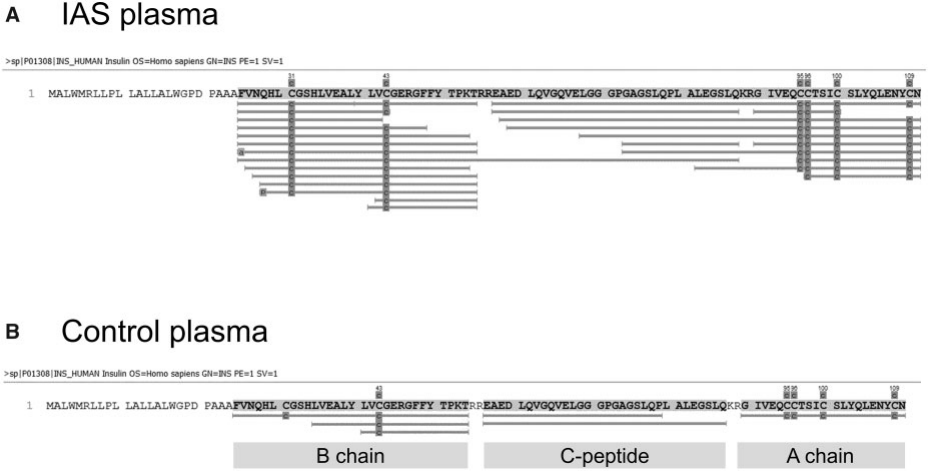
Plasma peptide matches to proinsulin in database search. Peptidomic analysis identified multiple peptides matching proinsulin, including full-length A and B chains and C-peptide, in insulin autoimmune syndrome plasma (A). A number of peptides spanning the B chain and C-peptide, and C-peptide and A chain, were detected, consistent with incomplete processing of the proinsulin molecule. Intact proinsulin could not be identified because the software was limited to peptides of up to 65 amino acids in length. Analysis identified peptides matching A and B chains and C-peptide in control plasma (B).

To identify plasma peptides with primary structural similarity to proinsulin, peptidomic analysis of the 6 IAS plasma samples with sufficient volume and 5 control plasma samples was undertaken. To detect both antibody-bound and free peptides, ACN was added to plasma to disrupt antibody–peptide interactions, and the samples reduced and alkylated to break disulfide bonds. Peptidomic analysis identified multiple peptides matching proinsulin, including fully processed A and B chains, and C-peptide. It also detected peptides spanning the B chain and C-peptide, and C-peptide and A chain (Fig. 3). For one sample with an extremely high concentration of antibody-bound insulin, 6 peptides spanning the C-peptide and A chain cleavage site were detected, in contrast to a single peptide spanning the B chain and C-peptide cleavage site (Fig. 3, A). B chain-C-peptide-cleavage site spanning peptides were not found in any other samples, and in control subjects, only fragments from fully processed prohormone were found (example shown in Fig. 3, B). The presence of intact proinsulin could not be examined because the bioinformatics software was limited to peptides of up to 65 amino acids in length.

### LC–MS Analysis of Intact Proinsulin-Derived Peptides in Plasma

To identify intact disulfide-bonded peptide hormones composed of the amino acid chains identified above (proinsulin, split/des proinsulins, and insulin), 6 IAS samples and 4 control samples were reextracted and analyzed, without reduction and alkylation, using high-throughput analysis. The molecular weights of proinsulin, des 31,32 proinsulin, and des 64,65 proinsulin were assigned as 9382, 9090, and 9116, respectively, and the theoretical *m/z* values of their [M + 5H]^5+^ to [M + 9H]^9+^ charge states were calculated. Extracted ion chromatograms were generated for these compounds and, in addition to insulin and C-peptide, proinsulin and des 31,32 proinsulin were identified (Supplemental Fig. 1), but des 64,65 proinsulin was not detected. Additional interrogation of the raw MS data of one IAS plasma sample identified a peptide with a molecular weight of 8964 Da, which eluted close to the des 31,32 proinsulin molecule, believed to be des 31–33 proinsulin (Supplemental Fig. 1, A). In contrast to IAS, only peaks representing fully processed insulin and C-peptide were detected in control plasma (Supplemental Fig. 1, B).

### LC–MS/MS Characterization of Intact Proinsulin-Derived Peptides in Plasma Extracts

To confirm the identity of the intact proinsulin/proinsulin-derived peptides, product ion spectra were generated and examined using high-throughput analysis. Because proinsulin and des proinsulin forms contain multiple disulfide bonds, their fragmentation patterns could not be matched using the software and manual matching was performed. Due to the large number of fragmentation events that occurred in C-peptide, identification of product ion spectra for des 31,32 and des 31–33 was possible (Supplemental Fig. 2). Proinsulin, in contrast, generated only a limited product ion spectrum containing multiple highly charged fragments whose source was not ascertained (Supplemental Fig. 3, A). Confirmatory matching was possible, however, following comparison of the product ion spectrum with a proinsulin reference standard (Supplemental Fig. 3, B). Des 31,32 and des 31–33 proinsulin standards could not be obtained. Des 64,65 proinsulin was not included in this targeted analysis since the peptide had not been detected in the characterization analysis using a full scan.

### Semiquantitative LC–MS/MS Analysis of Plasma Extracts

**Fig. 4.**
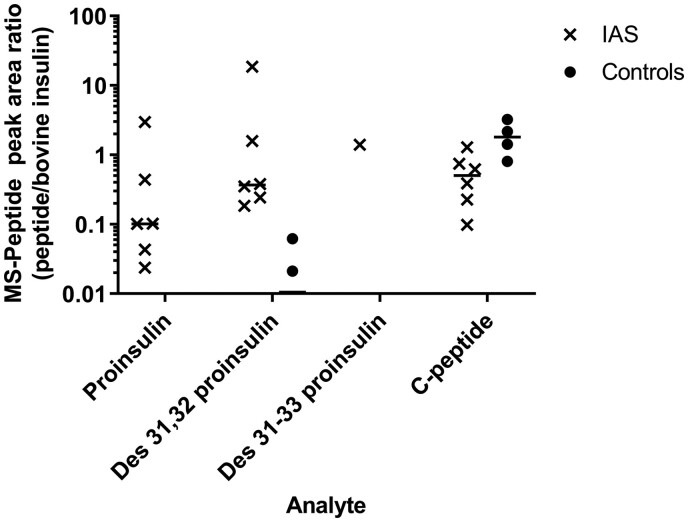
Relative abundance of proinsulin-derived peptides in insulin autoimmune syndrome (IAS). The mean (line) and individual data points (peak area ratios compared with bovine insulin internal standard) of proinsulin/proinsulin-derived peptides from 6 IAS plasma samples compared with 4 control samples. Data points below the limit of detection of the assay are not shown. MS, mass spectrometry.

Peptide peak area ratio values were generated by integrating the area under the curve using the Quan Browser software (Thermo Fisher Scientific). Proinsulin/proinsulin-derived peptide abundance was expressed as a ratio of the peak area to the bovine insulin internal standard. Unlike C-peptide, which appeared more abundant in control than IAS plasma, proinsulin and des 31,32 proinsulin were present at higher concentrations in IAS than in control plasma (Fig. 4). Des 31–33 proinsulin was detected in one IAS plasma sample. As expected, insulin was found to be more abundant in IAS than in control plasma (data not shown).

### IAS Plasma Analysis by Gel Filtration Chromatography with C-Peptide Immunoassay or LC–MS/MS

**Fig. 5.**
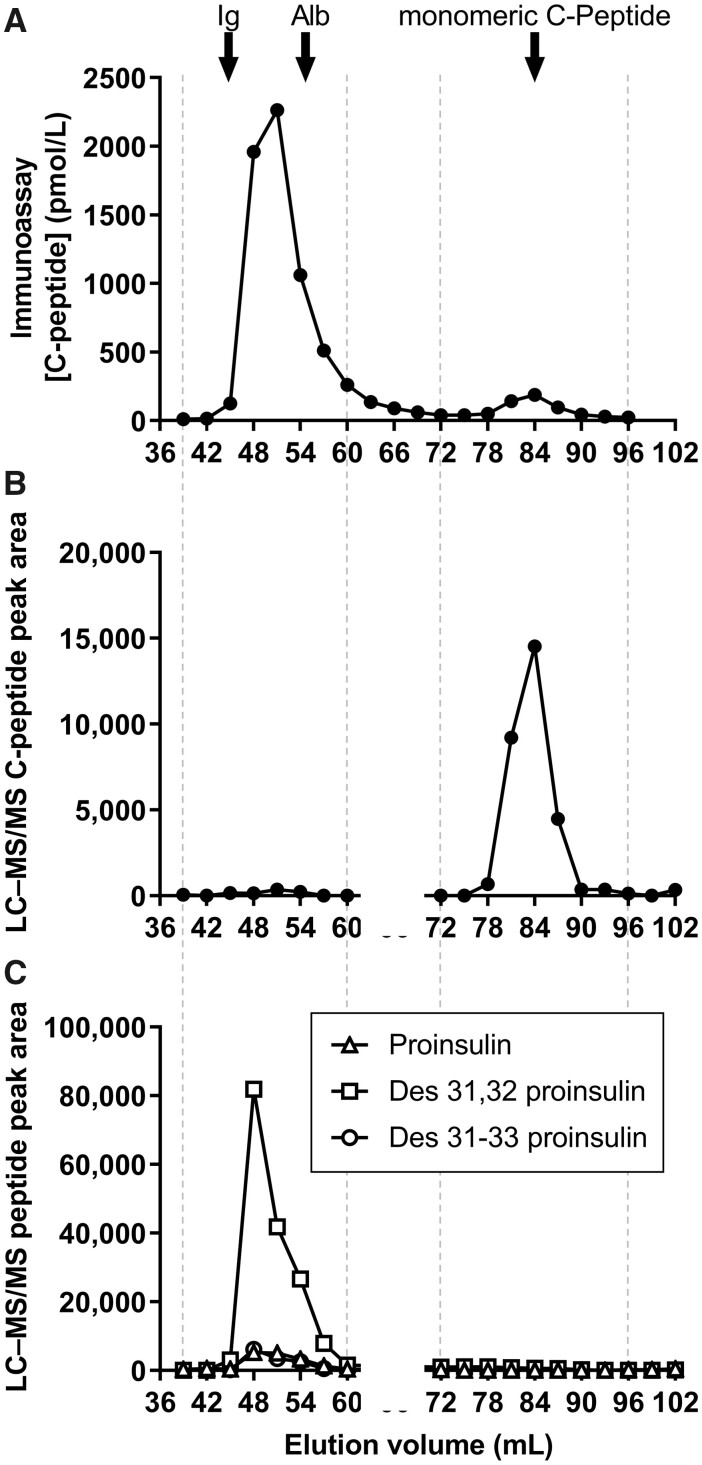
Size separation of proinsulin-derived peptides. Arrows show the elution volumes for immunoglobulin (Ig), albumin (Alb), and monomeric C-peptide. Immunoassay analysis demonstrated principally high molecular weight C-peptide immunoreactivity (A). GFC–LC–MS/MS analysis of the same plasma demonstrated only monomeric C-peptide (B), and high molecular weight proinsulin, des 31,32 proinsulin, and des 31–33 proinsulin (C).

Analysis of GFC-separated IAS plasma fractions by C-peptide immunoassay (Fig. 5), revealed 2 peaks: one large peak of HMW C-peptide immunoreactivity, and one much smaller peak consistent with monomeric C-peptide immunoreactivity (Fig. 5, A). In contrast, only monomeric C-peptide was demonstrated by LC–MS/MS (Fig. 5, B), refuting the presence of antibody-bound C-peptide in plasma. Small peaks of HMW proinsulin and HMW des 31–33 proinsulin, and a much larger peak of HMW des 31,32 proinsulin were identified, however (Fig. 5, C). LC–MS/MS results were consistent with the presence of antibody-bound proinsulin, des 31,32, and des 31-33 proinsulin in IAS plasma in the sample analyzed. These 3 peptides contain the C-peptide sequence, making cross-reactivity in the C-peptide immunoassay feasible. In-house data indicated proinsulin and insulin molar cross-reactivity of 120–140 and <0.01%, respectively, in the DiaSorin LIAISON XL C-peptide immunoassay (data not shown), consistent with the manufacturer’s and other independent observations (25–27). HMW insulin was demonstrated using immunoassay and LC–MS/MS, consistent with insulin-antibody complexes present in IAS (data not shown). GFC–C-peptide immunoassay and GFC–LC–MS/MS were repeated on another IAS plasma sample, and findings were broadly consistent with those above; data demonstrated HMW C-peptide immunoreactivity and MS HMW peaks for proinsulin, des 31,32 proinsulin, but no C-peptide (or des 31–33 proinsulin) (data not shown).

## Discussion

A disorder rarely reported in the West, IAS may be first suspected in the context of spontaneous hypoglycemia when a high insulin concentration and increased insulin/C-peptide molar ratio is identified (7). This profile may also occur in exogenous insulin administration (28), although very high C-peptide immunoreactivity will usually discriminate IAS from exogenous hyperinsulinemia. While suppression of C-peptide may be expected in response to exogenous insulin administration, the presence of substantial C-peptide immunoreactivity in the context of recurrent hypoglycemia in IAS has not been explained.

IAS diagnosis requires confirmation of circulating antiinsulin antibodies, but the presence of these is not specific for IAS (7). Available antiinsulin–antibody assays are, moreover, not standardized (29), and may be neither sufficiently sensitive (7) nor specific (5). There is thus clinical need to refine biochemical investigation of IAS, and MS may be incorporated into investigative algorithms as an orthogonal method that avoids the analytic vulnerabilities of immunoassay in the context of insulin autoimmunity.

C-peptide immunoassays can exhibit positive bias relative to MS in IAS (7), but this has not been explained. Suggested causes include antiinsulin–antibody complex interference (30), antibody binding to C-peptide or C-peptide and proinsulin (31), and immunoassay cross-reactivity of proinsulin immunocomplexes (10, 15). In one reported case of IAS, no endogenous-antibody binding of C-peptide was found (15).

PEG precipitation of plasma can identify hormone-immunoglobulin complexes, but is nonspecific and assay-dependent (5). In our study here, proportionally less C-peptide immunoreactivity remained following PEG precipitation of IAS plasma, compared with control plasma. Together with the GFC results from this study and from other reports (10, 15), this finding is consistent with HMW C-peptide immunoreactivity in IAS plasma, which is suggested to be an antibody-bound immunoreactive species. There was some overlap between IAS and control PPR values, and this may be attributable to sample heterogeneity in IAS: more monomeric C-peptide immunoreactivity, and/or less antibody-bound immunoreactive species (either a lower concentration or less immunoreactivity due to endogenous-antibody binding), may result in a PPR value closer to that of normal controls. It follows that use of C-peptide PPR may have only limited sensitivity in the investigation of suspected IAS, albeit a low PPR may be used as corroborative evidence.

LC–MS/MS analysis after organic solvent disruption of peptide–antibody bonds, and precipitation of large proteins (7), can establish total (unbound plus bound) peptide concentrations without the bias inherent in immunoassay. Using such a peptidomics approach, we have now clarified the nature of the immunocomplexes in IAS. No true C-peptide could be identified by MS analysis in the antibody-bound fractions. However, untargeted peptidomics detected multiple peptides spanning the proinsulin A chain and C-peptide, while targeted LC–MS/MS confirmed proinsulin and des 31,32 proinsulin at higher abundance in IAS plasma than in control plasma. In only one IAS patient was des 31–33 proinsulin detected, and further studies are required to determine whether this peptide is synthesized in vivo or is an artifact produced ex vivo. The higher proinsulin and des 31,32 proinsulin levels detected in IAS plasma than in control plasma is consistent with the prolonged half-life of these species, as well as that of insulin itself, in IAS (4, 32). Although proinsulin is highly unlikely to interfere to a clinically significant degree at normal proinsulin concentrations, it may thus be abundant enough in IAS to explain aberrant immunoassay C-peptide results. Although the reduced and alkylated peptide spanning the B chain and C-peptide was detected, abundance of intact des 64, 65 proinsulin in IAS plasma samples was insufficient to be detected using MS. In contrast to 64,65 proinsulin, the peptides proinsulin, des 31,32 proinsulin, and des 31–33 proinsulin, all have an intact link between the A chain and C-peptide (Fig. 1); however, a comparative study of antibody cross-reactivity using competitive radioligand binding (33) was precluded by lack of suitable standards for des proinsulin forms. Preferential processing of the 31/32 site before the 64/65 site has long been recognized (34), and even though the involvement of prohormone convertase 2 in the 64/65 processing has recently been challenged (35), a faster cleavage at the 31/32 site would make production and thus detection of des 64/65 insulin, which would be intact at the 31/32 site, less likely.

Accurate quantification of all proinsulin forms that have accumulated in plasma is required to determine whether these peptides potentially contribute to dysglycemia in IAS. C-peptide has not been shown to exhibit marked insulin-like activity (36, 37), but intact proinsulin (38), des 64,65 proinsulin, and des 31,32 proinsulin (39, 40) all have glucose-lowering activity. Indeed, proinsulin-secreting neuroendocrine tumors (41, 42) causing hypoglycemia with insulin concentrations below diagnostic thresholds (8) have been reported. It follows that antibodies with specificity for proinsulin may give rise to a subtype of IAS more challenging to discriminate. Further study of the kinetics/clearance of these antibody-bound proinsulin-derived peptides may offer further understanding of the pathophysiology of IAS.

## Supplemental Material

Supplemental material is available at *Clinical Chemistry* online.

## Supplementary Material

hvab043_Supplementary_Data

## Employment or Leadership

None declared.

## Consultant or Advisory Role

None declared.

## Stock Ownership

None declared.

## Honoraria

None declared.

## Expert Testimony

None declared.

## Patents

None declared.
